# What Instagram Means to Me: Links Between Social Anxiety, Instagram Contingent Self-worth, and Automated Textual Analysis of Linguistic Authenticity

**DOI:** 10.1007/s42761-024-00267-9

**Published:** 2024-09-13

**Authors:** Beatriz M. Brandao, Bryan T. Denny

**Affiliations:** Rice University, Department of Psychological Sciences, Houston, TX USA

**Keywords:** Linguistic authenticity, Contingent self-worth, Social anxiety, Social media, Instagram

## Abstract

While research has shown mixed effects of social media on mental health and well-being, little is known about the association between social media attitudes and objective measures of social interaction, such as linguistic authenticity. This study examined the relationship between self-reported social anxiety, linguistic authenticity, and Instagram contingent self-worth (ICSW). A total of 149 adults with active Instagram accounts completed online questionnaires and shared their Instagram comment data. Automated linguistic analysis of authenticity was performed on participants’ comment data using validated algorithms. Multiple linear regression showed that ICSW significantly moderated the relationship between social anxiety and linguistic authenticity, whereby higher levels of social anxiety marginally predicted lower linguistic authenticity at high levels of ICSW. As social media use continues to rise, this study emphasizes the need to explore the impact of social media interactions on emotional and social well-being.

**Supplementary Information:**

The online version contains supplementary material available at 10.1007/s42761-024-00267-9.

Social media has become an integral part of daily life. Instagram is one of the most popular photo and video sharing apps, with over 1 billion active monthly users (DataReportal, 2023). A growing body of research has shown that social media use (i.e., Facebook, Instagram, Twitter) is associated with a variety of negative effects on mental health and well-being, including increased anxiety and depression and lower self-esteem (Kelly et al., 2018). In addition, a systematic review found a strong positive correlation between Instagram use and depressive symptoms compared to non-users of Instagram and users of other social media (Adeyanju et al., 2021). However, findings have been mixed, with some studies indicating only weak or no links between social media use and health markers (Heffer et al., 2019). Nevertheless, these findings come with certain limitations, as most studies rely on coarse measures that ask individuals to report the frequency of their social media use, rather than delving into the specifics of how they engage in social media platforms. Little research has been conducted to explore the association between self-reported social anxiety, social media attitudes, and objective measures of social interaction, such as linguistic authenticity. This research aims to address this gap and shed light on the nuanced relationship between social media dynamics and psychological well-being.

Importantly, social media interactions take place within socially evaluative contexts, even though they are virtual. This context may affect highly socially anxious individuals more profoundly as they may be more motivated to make a specific impression to avoid negative evaluations. This tendency is in line with Schlenker and Leary’s Social Anxiety and Self-Presentation (SASP) model (Schlenker & Leary, 1982), which posits that people are more prone to suffer social anxiety when they are driven to produce a certain impression yet are unsure of their capacity to do so. Social anxiety, particularly social anxiety disorder (SAD), involves a persistent awareness and fear of being judged by others. This pervasive fear can lead to behaviors aimed at tightly controlling one’s self-presentation to align with perceived social expectations. Such behaviors may be a direct manifestation of inauthenticity, as they involve altering one’s expressions to better fit social norms rather than conveying genuine thoughts or feelings.

Therefore, the link between social anxiety and authenticity on social media is based in the core dynamics of how socially anxious individuals perceive and navigate social evaluations. The motivation to manage impressions, driven by fears of negative evaluation, makes the study of authenticity particularly relevant in the context of social anxiety. Thus, it is essential to explore how social anxiety predicts the authenticity of self-presentations on social media, which can have profound implications for mental health.

In line with this concept, a recent study examined how people portray and control their self-image on Instagram and found that people’s levels of social anxiety were related to the approval of other Instagram users as part of their sense of worth (Lopez & Polletta, 2021). Contingent self-worth, distinct from self-esteem, refers to individuals’ perception of what they need to do, or how they need to act, to attain value and personal worth (Crocker & Knight, 2005). While this construct can manifest in various life domains—such as career achievements or academic success—it also extends to social media. In particular, the use of social media has been shown to influence contingent self-worth, where behaviors aimed at gaining social approval are associated with fluctuations in self-esteem and could heighten the risk of mental health disorders, such as depression (Crocker, 2002).

Given the prevalent role of social media in modern social interactions, we focused on Instagram—a platform characterized by its visual and interactive nature, which may uniquely impact contingent self-worth. This choice is supported by findings from Lopez and Polletta (2021), which demonstrated how users’ sense of worth on Instagram, defined as Instagram contingent self-worth (ICSW), is significantly impacted by the approval from other users. This particular form of contingent self-worth involves the value and self-esteem individuals derive from their engagement on Instagram, including the number of likes and comments received on a post, which are more pronounced among those with higher levels of social anxiety. The relevance of Instagram in the context of contingent self-worth is further highlighted by its distinct user engagement compared to other platforms, which centers around visual self-presentation and instant feedback. Thus, our study aims to explore how these unique aspects of Instagram influence intrapersonal processes, particularly for individuals with high social anxiety, and how this, in turn, affects their interactions on the platform.

Within this evolving research landscape, there is a growing trend that leverages social media data as a powerful tool for predicting not only mental health outcomes but also a wide array of psychological constructs. Among these constructs, authenticity stands out as a multifaceted concept with roots extending from philosophy to psychology. Specifically, in psychology, Barrett-Lennard’s, 1998 model delineates authenticity into three distinct yet interconnected components: self-alienation, which describes the disconnect between an individual’s true self and their expressed feelings and thoughts; authentic living, which measures the congruence between an individual’s actions and their core values; and acceptance of external influence, which assesses the impact of external pressures on personal values and behaviors.

These theoretical components of authenticity are crucial for understanding the dynamics within social media interactions. Authenticity, as defined and operationalized in our study, specifically relates to the extent to which individuals express their genuine self in online settings. This focus is informed by a substantial body of empirical research that underscores the significance of authenticity in psychological well-being. Studies have consistently shown a positive correlation between authenticity and key markers of psychological health such as subjective well-being and self-esteem (Goldman & Kernis, 2002; Theran, 2010; Wenzel & Lucas-Thompson, 2012). Conversely, the lack of authenticity, characterized by high levels of self-alienation, has been associated with adverse outcomes, including dysfunctional thought processes and an increased susceptibility to stress and psychological distress (Akin & Akin, 2014; Satici et al., 2013).

Other work has also examined associations between self-reported authenticity and the utilization of emotion regulation strategies, like expressive suppression and cognitive reappraisal (English & John, 2013). Expressive suppression refers to the inhibition or reduction of emotion expressive behavior, whereas cognitive reappraisal refers to changing the way one thinks about an emotional stimulus. English and John found that individuals who engage in expressive suppression were more prone to experience feelings of inauthenticity, while no association was found with the use of cognitive reappraisal. This body of work collectively highlights the important role of authenticity in predicting psychological well-being and motivates further exploration of authenticity within the context of social media interactions.

## Present Study

The present study was designed to probe the relationship between social anxiety and objective measures of linguistic authenticity in social media interactions, including examination of Instagram contingent self-worth (ICSW) as a potential moderator. We predicted that participants with higher social anxiety and greater use of expressive suppression would exhibit lower levels of linguistic authenticity on Instagram. Additionally, we predicted that the relationship between social anxiety, expressive suppression, and linguistic authenticity would be moderated by ICSW, such that individuals with greater ICSW would show a stronger negative relationship between social anxiety and linguistic authenticity as well as expressive suppression and linguistic authenticity.

## Method

### Participants

An a priori power analysis using G*Power version 3.1.9.7 (Faul et al., 2007) determined that a sample size of 108 was needed to achieve 90% power by specifying a medium effect size (*f*^2^ = .10), and alpha = .05, for a two-sided test. To ensure sufficient power, we recruited 200 participants from the Rice University psychology participant pool. After excluding participants who did not finish the study (i.e., decided to not share their Instagram data, did not have comment data), our final sample consisted of 149 adults. The inclusion criteria consisted of participants being at least 18 years old, being able to read and write in English, having an Instagram account, and having actively used Instagram for the past year. Participants were excluded if they were not 18 years old or did not have or use Instagram. Participants were compensated with course credit. All participants provided informed consent in accordance with guidelines set by the Institutional Review Board at Rice University.

### Procedure

Participants completed the Social Anxiety Questionnaire (Caballo et al., 2012), Difficulties in Emotion Regulation Questionnaire (Kaufman et al., 2016), Emotion Regulation Questionnaire (Gross & John, 2003), and Instagram Contingent Self-Worth Questionnaire (Lopez & Polletta, 2021) through online surveys. Participants then received instructions to retrieve their Instagram comment history data. Detailed instructions for retrieval are provided in Supplementary Information. The comment file contained the history of all comments participants have made on Instagram posts or/and Instagram stories of other users. Then after cleaning the data, we performed automated linguistic analysis of authenticity using validated and standardized algorithms through Linguistic Inquiry and Word Count (LIWC) software (Boyd et al., 2022), as described in greater detail below.

### Data Cleaning

In the process of preparing our dataset for analysis, we implemented several criteria to filter and remove specific types of comments: comments that did not contain English words, those consisting solely of symbols or emoticons (e.g., emojis were ignored by the algorithms), and comments with identical content (duplicates). Additionally, any comments featuring identifiable usernames or name tags were also removed. This data cleaning approach was employed by three trained coders to ensure that our subsequent analyses would consist of original English language content. The final dataset contained 53,259 comments with a total word count of 319,589. For further details, please refer to Supplementary Information.

### Measures

#### Emotion Regulation

Emotion regulation was measured by the Emotion Regulation Questionnaire (Gross & John, 2003), which is a 10-item scale designed to measure respondents’ tendency to regulate emotions in two ways: (1) cognitive reappraisal (six items; items 1, 3, 5, 7, 8, and 10) and (2) expressive suppression (four items; items 2, 4, 6, and 9). Respondents answer each item on a 7-point Likert-type scale ranging from 1 (strongly disagree) to 7 (strongly agree). The higher the score, the higher the frequency of using the emotion regulation strategy. The internal consistency (Cronbach’s *α*) was .825.

#### Difficulties in Emotion Regulation

The Difficulties in Emotion Regulation Scale (DERS) is a well-validated and widely used self-report measure for assessing emotion regulation problems among adolescents and adults. We use the short form of DERS. DERS-SF is an 18-item measure used to identify adult emotional regulation issues (Kaufman et al., 2016). This measure covers 4 dimensions of emotional regulation: (1) awareness and understanding of emotions, (2) acceptance of emotions, (3) the ability to engage in goal-directed behavior and refrain from impulsive behavior when experiencing negative emotions, and (4) access to emotion regulation strategies perceived as effective. Scale internal consistency, Cronbach’s alpha = .94.

#### Social Anxiety

The Social Anxiety Questionnaire measures social anxiety for Adults (SAQ-A), which consists of 30 items in which participants are asked to rate their level of “unease, stress, or nervousness” in different social situations using a 5-point Likert scale ranging from 1 (“Not at all or very slight”) to 5 (“Very high or extremely high”). Items follow a five-factor structure: (1) speaking in public/talking with people in authority (e.g., “Talking to a superior or a person in authority”), (2) interactions with the opposite sex (e.g., “Being asked out by a person I am attracted to”), (3) assertive expression of annoyance, disgust, or displeasure (e.g., “Having to ask a neighbor to stop making noise”), (4) criticism and embarrassment (e.g., “Being criticized”), and (5) interactions with strangers (e.g., “Attending a social event where I know only one person”) (Caballo et al., 2012). The responses to each item are added together to give each participant a single score that represents their propensity to experience social anxiety. Scale internal consistency, Cronbach’s alpha = .95.

#### Instagram Contingent Self-worth

This scale was retrieved from Lopez and Polletta (2021). It is an adaptation from the Contingencies of Self-Worth Scale (Crocker et al., 2003), specifically from the subscale that represents contingent self-worth based on others’ approval. Using a 5-point Likert scale (1 = strongly disagree; 2 = disagree; 3 = neutral; 4 = agree; 5 = strongly agree), participants are asked to rate their level of agreement with the following four statements: (1) “When I get a lot of likes and new followers on my Instagram, my self-esteem increases”; (2) “I feel worthwhile when others like or comment on my Instagram posts”; (3) “When my Instagram posts or comments go unnoticed, I feel badly about myself”; and (4) “My self-esteem depends on how popular and active my Instagram profile is.” Scale items showed Cronbach’s alpha of .80.

#### Authenticity Composite

The Linguistic Inquiry and Word Count (LIWC) is a software program that analyzes text for instances of particular words and terms to determine the extent to which different categories are used in that text. For the authenticity composite, LIWC uses terms that convey honesty and genuineness derived from previous empirical studies to create a percentile summary score (Boyd et al., 2022). Dimensions that positively load onto the authenticity score include self-references (e.g., I), insight words (e.g., aware), differentiation words (e.g., but), and relativity terms (e.g., above). Dimensions that negatively load onto the authenticity index include discrepancies from reality (e.g., must) and third-person singular pronouns (she-he words) (e.g., she) (Markowitz et al., 2022). The authenticity score ranges from 0 (low authenticity) to 100 (high authenticity). The measure is calculated based on the formula, i + insight + differ + relativ − discrep − shehe (Jordan et al., 2018). Higher values are related to a more honest, personal, and disclosing communication style, and lower values imply a more circumspect, distant communication style (Pennebaker et al., 2015). Please see Supplementary Information for additional details.

### Analysis

We first computed pairwise correlations between the variables of interest [i.e., social anxiety (reflected by SAQ-A scores), emotion regulation (expressive suppression and cognitive reappraisal), difficulties in emotion regulation (DERS), Instagram contingent self-worth, linguistic authenticity, and word and comment count]. All continuous variables were centered, and categorical variables such as gender were dichotomized into female vs. non-female, and race/ethnicity was categorized as white, non-Hispanic vs. non-white, non-Hispanic. Next, we used Jamovi version 2.2.5 to specify multiple linear regression models with social anxiety and emotion regulation tendencies predicting linguistic authenticity. To assess different aspects of emotion regulation, we used both DERS and ERQ. The ERQ primarily measures the frequency of employing two specific emotion regulation strategies: expressive suppression and cognitive reappraisal. In contrast, DERS assesses multiple dimensions of emotion regulation difficulties, providing a broader metric of how individuals typically experience and respond to emotional situations. Due to the high correlation between DERS and social anxiety (*p* < .001), DERS was excluded from the primary regression analysis to avoid multicollinearity issues. We focused on expressive suppression in our primary analysis, consistent with our hypothesis and previous findings suggesting its association with authenticity (English & John, 2013). As part of our exploratory analyses, we also included models incorporating cognitive reappraisal as a moderation term and models without any emotion regulation covariates (Supplementary Tables 2 and 3). We performed these analyses to examine the robustness of our findings across different dimensions of emotion regulation and to explore potential interactions that may not be captured by focusing solely on one emotion regulation tactic (i.e., expressive suppression). Significant interactions were then further analyzed using the PROCESS module in SPSS to examine Johnson-Neyman intervals, which calculates the values of the moderator along a continuum to determine the point at which the moderator begins influencing the outcome variable (Hayes, 2013). Participants’ age, sex, race/ethnicity, daily Instagram screen time, number of followers, and word and comment count were included as covariates in all models.

## Results

Table 1 provides descriptive statistics for all variables. We also assessed daily time spent on Instagram: 36.3% of participants reported spending 0–30 min, 38.2% reported 30–60 min, 18.7% reported 60–90 min, 3.3% reported 90–120 min, and 3.5% reported more than 120 min.

**Table 1:** Descriptive statistics and sample characteristics

Variable	Mean ± SD	*N* (%)
Social anxiety (SAQ scores)	91.5ª ± 21.7	–
Expressive suppression (ERQ)	15 ± 5.23	–
Cognitive reappraisal (ERQ)	28.6 ± 5.73	–
Instagram contingent self-worth	11.9 ± 3.07	–
DERS	43.7 ± 13	–
Linguistic authenticity	26.6 ± 9.8	–
Comment count	357 ± 779	–
Word count	6.6 ± 2.64	–
Followers	867 ± 534	–
Age	19.3 ± 1.13	–
Male	–	43 (28.9)
Female	–	106 (71.1)
Asian	–	72 (48.32)
White-Caucasian	–	29 (19.46)
Black or African American	–	15 (10.07)
Hispanic or Latino	–	14 (9.40)
More than one race	–	19 (12.75)

^ª^High social anxiety > 90 (51.01%) (Caballo et al., 2012)

Next, we conducted pairwise correlations among all variables of interest. As expected, social anxiety was positively associated with use of expressive suppression, *r* = .319 (95% CI .456, .167), *p* < .001, difficulties in emotion regulation, *r* = .623 (95% CI .712, .513), *p* < .001, and negatively associated with use of cognitive reappraisal, *r* = .267 (95% CI .110, .410), *p* = .001. Additionally, there was a significant positive relationship between Instagram contingent self-worth and difficulties in emotion regulation (DERS), *r* = .168 (95% CI .320, .008), *p* = .040, and a negative relationship between comment count and expressive suppression, *r* =  − .237 (95% CI − .079, − .383), *p* = .004. See Table 2 for all pairwise correlations.

**Table 2:** Pairwise correlations between measures of interest

Variable	1	2	3	4	5	6	7
1. Social anxiety (SAQ scores)							
2. Expressive suppression (ERQ)	.319***						
3. Cognitive reappraisal (ERQ)	− .267**	− .036					
4. Instagram contingent self-worth	.123	− .069	− .117				
5. DERS	.623***	.394***	− .315***	.168*			
6. Linguistic authenticity	.037	.081	− .018	.091	.068		
7. Word count	.076	− .035	− .070	− .022	.186*	− .014	
8. Comment count	− .105	− .237**	.033	.058	.067	.057	− .117

*p < .05, **p < .01, ***p < .001

We next examined a multiple regression model incorporating social anxiety (SAQ scores) and expression suppression and cognitive reappraisal (ERQ) as well as age, gender, race/ethnicity, Instagram daily use, number of Instagram followers, and comment and word count in predicting linguistic authenticity. This model satisfied all assumptions for multiple regression analysis. The variance inflation factor (VIF) for each predictor was low, with values below 2. The detailed results of this regression analysis are presented in Supplementary Table 1. Results revealed that social anxiety, as assessed by SAQ scores, did not significantly predict linguistic authenticity (*b* = .01, *p* = .759). Similarly, neither expression suppression nor cognitive reappraisal usage emerged as a significant predictor of linguistic authenticity (*b* = .12, *p* = .481, *b* =  − .03, *p* = .839, respectively). These results indicated that, overall, social anxiety as well as expressive suppression and cognitive reappraisal usage tendencies were not significantly associated with linguistic authenticity. That said, we were interested to probe whether such relationships may be moderated by individuals’ levels of Instagram contingent self-worth.

Thus, as planned, we added Instagram contingent self-worth (ICSW) to an additional multiple regression model as well as each covariate from the previous model (i.e., age, gender, race/ethnicity, Instagram daily use, number of Instagram followers, comment and word count) to assess whether ICSW moderated the relationship between social anxiety and linguistic authenticity, as well as tendencies to engage in expressive suppression. Exploratory models also incorporating cognitive reappraisal as a moderation term, or without any emotion regulation tendency covariates, are given in Supplementary Tables 2 and 3. This model satisfied all assumptions for multiple regression analysis. The variance inflation factor (VIF) for each predictor was low, with values below 2. Regression results for this model are shown in Table 3.

**Table 3:** Multiple regression results including moderation terms predicting linguistic authenticity

Predictors	*B*	*SE*	*Lower 95% CI*	*Upper 95% CI*	*T*	*p*
(Intercept)	22.88	14.45	− 5.7	51.47	1.58	.116
Social anxiety (SAQ)	0.00	0.04	−0 .08	0.08	0.17	0.987
Expressive suppression	0.17	0.18	− 0.17	0.52	0.98	0.324
Age	0.34	0.74	− 1.11	1.80	0.46	0.642
Gender	2.25	1.91	− 1.51	6.02	1.18	0.238
Race/ethnicity	− 1.01	1.79	− 4.55	2.51	− 0.56	0.570
Instagram daily time	−0 .25	0.41	− 1.07	0.56	− 0.62	0.537
Followers	−0 .00	0.00	−0 .00	0.00	− 1.53	0.128
Word count	0.01	0.31	− 0.61	0.63	0.03	0.972
Comment count	0.00	0.00	−0 .00	0.00	1.50	0.135
ICSW	0.46	0.28	−0 .09	1.01	1.65	0.102
Social anxiety × ICSW	−0 .03	0.01	−0 .06	0.00	− 2.36	0.019*
Expressive suppression × ICSW	0.09	0.05	−0 .01	0.21	1.72	0.086

*p < .05

**Fig. 1:**
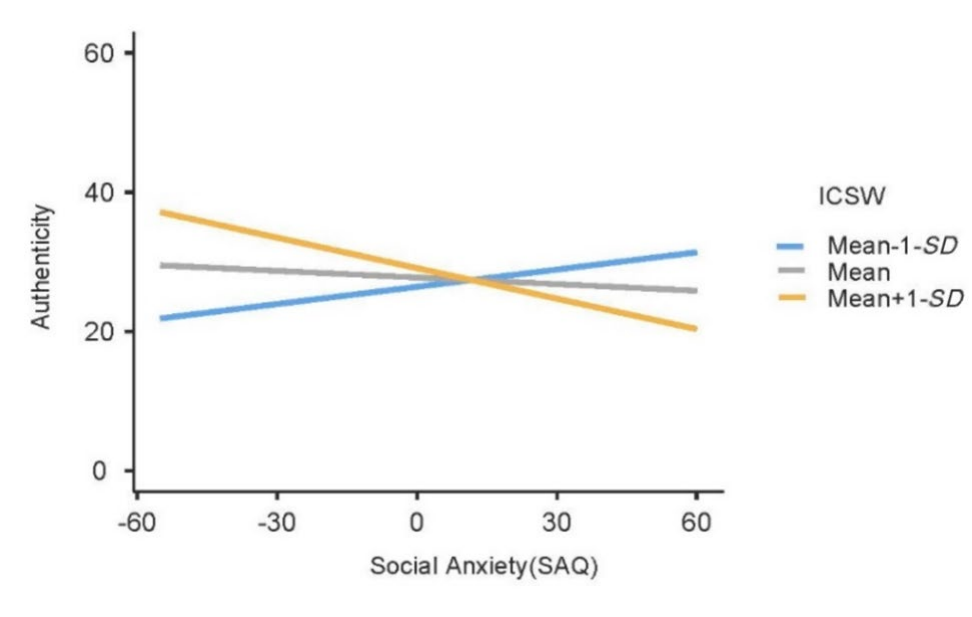
Interaction between Instagram contingent self-worth (ICSW) and social anxiety predicting linguistic authenticity

The interaction between expressive suppression and ICSW was marginally significant (*b* = .09, *p* = .086), and an analysis of simple slopes further revealed no significant effects for this interaction (all *p* > .10). The interaction between social anxiety and ICSW was significant (*b* =  − .03, *p* = .019). An analysis of simple slopes indicated that at one standard deviation below the mean level of ICSW, the estimated effect of social anxiety on linguistic authenticity was not statistically significant (*b* = .18, *t*(135) = 1.31, *p* = .192, 95% CI − .042, .207). At the mean level of ICSW, the estimated effect was not significant (*b* =  − .05, *t*(135) =  − .48, *p* = .628, 95% CI − .134, .081). At one standard deviation above the mean ICSW, the effect is marginally significant (*b* =  − .29, *t*(135) =  − 1.77, *p* = .078, 95% CI − .287, .015) (Fig. 1).

**Fig. 2:**
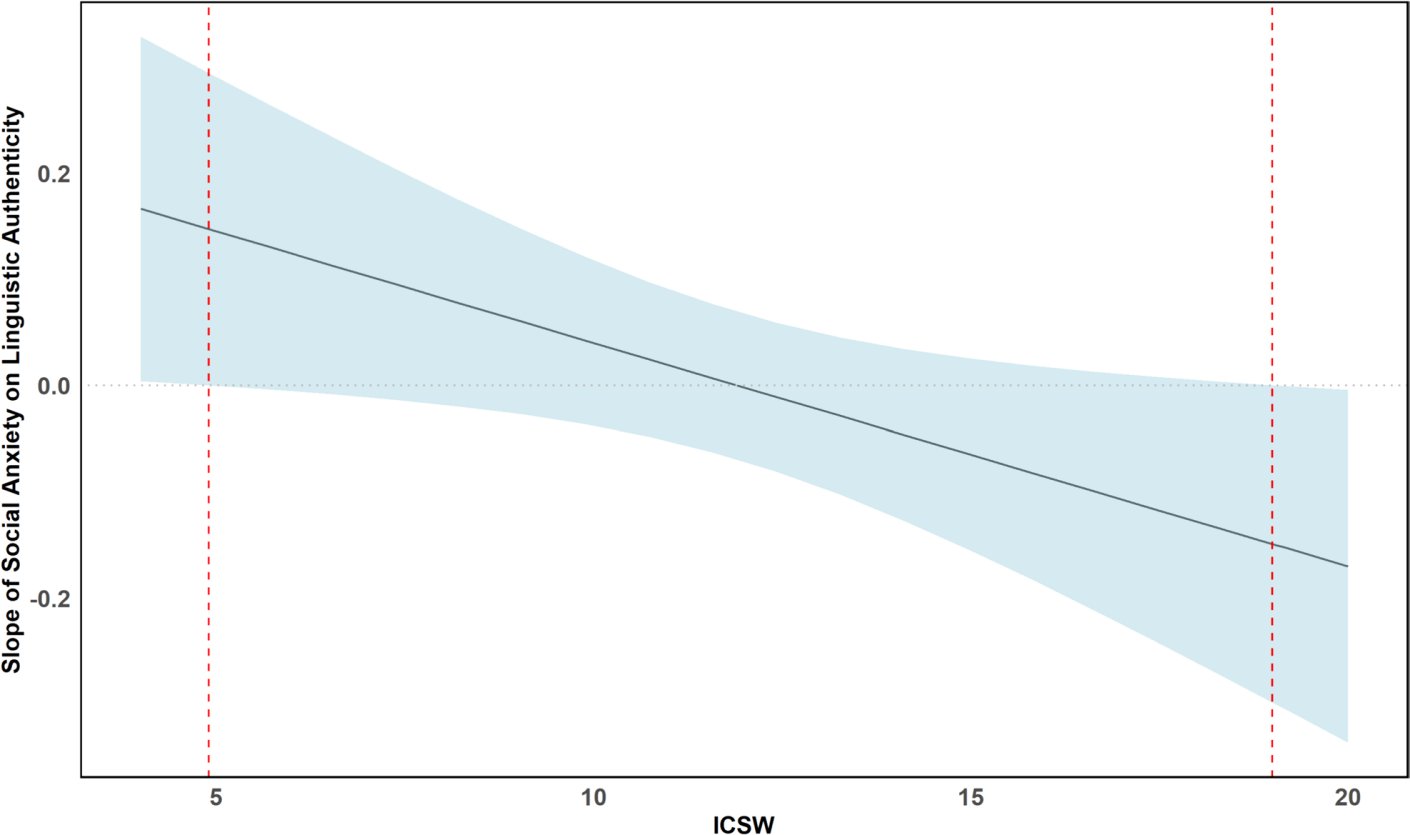
Johnson-Neyman interaction plot for the conditional relation between social anxiety and linguistic authenticity as a function of Instagram contingent self-worth (ICSW). The figure displays the simple regression line (black) representing the effect regressed on ICSW and its 95% confidence bands (blue shaded area). The vertical red dotted lines represent the boundary of the regions of significance

To probe the interaction further, we conducted a Johnson-Neyman analysis, which indicated specific points along the ICSW continuum where the association between social anxiety and linguistic authenticity becomes statistically significant. Specifically, significant effects were observed at ICSW values of 4.9 and 19.0, representing low and high levels of ICSW, respectively. These values fall within the observed range of ICSW scores in our dataset (Fig. 2).

## Discussion

This study investigated associations between social anxiety, expressive suppression, and linguistic authenticity of Instagram comments, as well as the moderating effect of Instagram contingent self-worth (ICSW). Multiple regression revealed that the relationship between social anxiety and linguistic authenticity was moderated by ICSW. Simple slopes analysis showed a marginally significant effect at high ICSW levels, such that greater social anxiety was associated with lower linguistic authenticity. A Johnson-Neyman analysis identified significant effects at specific ICSW thresholds. When ICSW was high, higher social anxiety was associated with lower linguistic authenticity. Conversely, when ICSW was low, higher social anxiety was associated with higher linguistic authenticity; however, this latter effect in the Johnson-Neyman analysis should be interpreted cautiously given that the simple slope was not significant. Overall, these findings support our hypothesis, suggesting that social anxiety levels may relate to a sense of self-worth linked to specific aspects of Instagram usage, ultimately relating to how authentic individuals seem on the platform.

The results also showed that the main effect of social anxiety as well as expressive suppression predicting linguistic authenticity on social media was not significant. This finding contrasts with previous research that has found a negative association between self-reported authenticity and the use of expressive suppression and social anxiety (English & John, 2013; Plasencia et al., 2011). These discrepancies might be attributed to the differences in measurement approaches: previous studies used subjective self-report methods to assess authenticity, whereas our study employed automated linguistic measures. Consistent with English and John, we did not find a significant relationship between cognitive reappraisal usage and authenticity.

Overall, our findings offer empirical support to the Schlenker and Leary’s Social Anxiety and Self-Presentation (SASP) model (Schlenker & Leary, 1982). The SASP model suggests that people tend to feel increased social anxiety when they are driven to create a particular impression but doubt their capability to do so. The inclination to present oneself in a manner consistent with perceived social expectations may become even more pronounced in the high stakes, socially evaluative context of social media. In the present work, this inclination to curate a particular self-image may be leading people to change the way they interact in Instagram comments, making them less authentic. Particularly, those more prone to social anxiety, and who place more value on how they are seen on Instagram, may feel an increased need to carefully curate their online presentation, making their interactions on social media less authentic. This in turn may contribute to creating even more feelings of social anxiety.

One of the strengths of this study is that it used an objective measure of authenticity through linguistic analysis. This approach provides a more impartial and less biased understanding of an individual’s authenticity levels in their online interactions. Moreover, the study highlights the importance of examining moderating factors that may better explain the association between emotional processes and social media use. By investigating social anxiety and emotion regulation tendencies and their relationship with authenticity and Instagram contingent self-worth, the study attempted to capture several aspects of people’s experiences with Instagram.

It should be noted that the correlational nature of the study limits the ability to draw causal conclusions from the data. The results do not indicate whether social anxiety causes changes in Instagram contingent self-worth and linguistic authenticity or whether or not these factors are influenced by other variables. It is possible that the relationship between social anxiety, self-worth, and authenticity is bidirectional or that there are other unknown factors at play. Additionally, as our focus was specifically on Instagram, the findings may not necessarily apply to other social media platforms, where user interaction dynamics and content presentation can differ significantly. Future research utilizing experimental or longitudinal designs will be important to better understand the causal relationships between these variables. Moreover, extending these studies across different social media platforms could provide insights into whether the observed associations are consistent or vary according to the platform-specific features and user engagement styles.

Overall, the findings suggest that Instagram contingent self-worth may play a role in shaping the relationship between social anxiety and authenticity when using the platform. However, further research is needed to explore the causal relationships between these variables and identify effective interventions that may promote healthy social media use. As social media use continues to rise among adolescents and other vulnerable populations, who are prone to social anxiety and constant comparison, it is crucial to explore the interplay between social media use and indicators of emotional and social well-being. The findings from this and related studies may ultimately be helpful for healthcare providers in assessing the potential negative effects of social media use on a person’s emotional and psychological well-being by considering whether and how their self-worth is contingent on using the social media itself.

## Supplementary Information

Below is the link to the electronic supplementary material.

Supplementary file1 (DOCX 44 KB)

## Additional Information

### Availability of data and material

The datasets analyzed are available in the Open Science Framework repository at https://osf.io/5ad2t/?view_only=71081c2d95e141ce8dba6fd26baaab11.
