# Supplementary material for: What Instagram Means to Me: Links Between Social Anxiety, Instagram Contingent Self-worth, and Automated Textual Analysis of Linguistic Authenticity
Source: Affect Sci. 2024 Sep 13;5(4):449–57. doi: 10.1007/s42761-024-00267-9 (PMC11624144; doi:10.1007/s42761-024-00267-9)
Supplement: Supplementary file 1 — Supplementary file1 (DOCX 44 KB) [file 42761_2024_267_MOESM1_ESM.docx]

**Supplementary Information**

**Article title:** What Instagram means to me: Links between social anxiety, Instagram contingent self-worth, and automated textual analysis of linguistic authenticity

**Journal:** Affective Science

**Authors:** Beatriz M Brandao^1^ and Bryan T Denny^1^

*^1^Rice University, Department of Psychological Sciences, Houston, TX, United States*

**Corresponding author:** beatriz.brandao@rice.edu (Beatriz M Brandao)

**LIWC Authenticity Index Formula**

The following formula is used to calculate the LIWC authenticity index (Jordan et al., 2018). All variables described below are LIWC-22 default variables.

i + insight + differ + relativ – discrep – shehe

LIWC2022 automatically scores this index where 100 = high on authenticity, 0 = low on authenticity, and 50 = middling authenticity. The measure is calculated based on the formula above, and “then converted to percentiles based on standardized scores from large comparison corpora” (see Boyd et al. 2022). The authenticity index is a bottom-up composite measure that stems from seminal work on deception and language research. For example, prior work had participants write about their honest and false feelings about a range of topics (e.g., abortion, their friends) (Newman et al., 2003). The language categories in the authenticity index are the result of factor-analyzing such texts and distinguishing false from truthful speech.

**Examples of High and Low Authenticity Texts**

**Study Data: Instagram comments**

“I’m sorry I can’t help myself ok” (Authentic score: 99)

“Yes, Karen is among the women I respect, but I respect all women equally because that’s how you respect women” (Authentic score: 98)

“Drink milk to grow big and strong” (Authentic score: 24.32)

**Instagram Data Cleaning**

1. **Comments collection procedure**

All comments were downloaded by participants from their personal Instagram account. Participants received the following instructions:

1. Login on to your Instagram profile from a computer (not your phone).
2. Click your profile picture in the top right, then click 
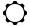
**Settings**.
3. Click **Privacy and security**.
4. Scroll down to **Data download** and click **Request download**.
5. Enter the email address where you'd like to receive a link to your data.
6. Click 
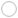
next to **JSON** to select the format to receive your data in, then click **Next**.
7. Enter your Instagram account password and click **Request download**.
8. You'll soon receive an email titled **Your Instagram data** with a link to your data.
9. Click **Download data** and follow the instructions to finish downloading your information.
10. Please select only your **comments file** and upload to link below.

The comments file contained the history of all comments participants have made on Instagram posts or/and Instagram stories.

1. **Comments exclusions/cleaning**

Comments meeting the following criteria were removed from the dataset:

- comments with non-English words
- comments that only contained symbols or emoticons
- comments with identifiable usernames or name tags
- comments with identical content

We followed this data cleaning procedure in an attempted to focus our analyses on comments with original, English language content that could be calculated by the LIWC authenticity index.

**Supplemental Results**

Supplemental Table 1. Multiple regression results predicting linguistic authenticity.

| Predictors | B | SE | *Lower 95% CI* | *Upper 95% CI* | t | *p* |
| --- | --- | --- | --- | --- | --- | --- |
| (Intercept) | 21.24 | 14.74 | -7.91 | 50.40 | 1.41 | 0.152 |
| Social anxiety (SAQ) | 0.01 | 0.04 | -0.07 | 0.10 | 0.30 | 0.759 |
| Expressive suppression | 0.12 | 0.17 | -0.22 | 0.47 | 0.70 | 0.481 |
| Cognitive reappraisal | -0.03 | 0.15 | -0.32 | 0.26 | -0.20 | 0.839 |
| Age | 0.37 | 0.75 | -1.19 | 1.86 | 0.49 | 0.622 |
| Gender | 2.61 | 1.93 | -1.21 | 6.43 | 1.34 | 0.179 |
| Race/Ethnicity | -0.63 | 1.81 | -4.21 | 2.95 | -0.35 | 0.728 |
| Instagram daily time | -0.18 | 0.41 | -1.01 | 0.64 | -0.43 | 0.667 |
| Followers | -0.00 | 0.00 | -0.00 | 0.00 | -1.12 | 0.265 |
| Word count | -0.06 | 0.31 | -0.69 | 0.56 | -0.20 | 0.842 |
| Comments count | 0.00 | 0.00 | -0.00 | 0.00 | 1.38 | 0.170 |

Supplemental Tabe 2. Multiple regression results including ICSW as moderation term predicting linguistic authenticity.

| Predictors | B | SE | *Lower 95% CI* | *Upper 95% CI* | t | *p* |
| --- | --- | --- | --- | --- | --- | --- |
| (Intercept) | 22.16 | 14.45 | -6.40 | 50.74 | 1.53 | 0.127 |
| Social anxiety (SAQ) | 0.01 | 0.03 | -0.06 | 0.09 | 0.39 | 0.692 |
| ICSW | 0.42 | 0.27 | -0.12 | 0.97 | 1.52 | 0.131 |
| Age | 0.37 | 0.73 | -1.07 | 1.83 | 0.51 | 0.609 |
| Gender | 2.81 | 1.86 | -0.87 | 6.50 | 1.51 | 0.133 |
| Race/Ethnicity | -0.63 | 1.77 | -4.13 | 2.87 | -0.35 | 0.722 |
| Instagram daily time | -0.25 | 0.41 | -1.07 | 0.57 | -0.60 | 0.545 |
| Followers | -0.00 | 0.00 | -0.00 | 0.00 | -1.35 | 0.180 |
| Word count | -0.09 | 0.31 | -0.71 | 0.51 | -0.31 | 0.753 |
| Comments count | 0.00 | 0.00 | -0.00 | 0.00 | 1.17 | 0.243 |
| Social anxiety x ICSW | -0.01 | 0.01 | -0.04 | 0.00 | -1.65 | 0.101 |

Supplemental Table 3. Multiple regression results including ERQ subscales as moderation terms predicting linguistic authenticity.

| Predictors | B | SE | *Lower 95% CI* | *Upper 95% CI* | t | *p* |
| --- | --- | --- | --- | --- | --- | --- |
| (Intercept) | 22.61 | 14.81 | -6.68 | 51.91 | 1.52 | 0.129 |
| Social anxiety (SAQ) | 0.00 | 0.04 | -0.08 | 0.09 | 0.06 | 0.949 |
| Expressive suppression | 0.17 | 0.17 | -0.17 | 0.53 | 0.99 | 0.323 |
| Cognitive reappraisal | 0.02 | 0.15 | -0.27 | 0.32 | 0.15 | 0.877 |
| Age | 0.32 | 0.74 | -1.15 | 1.80 | 0.43 | 0.664 |
| Gender | 2.30 | 1.95 | -1.56 | 6.18 | 1.17 | 0.241 |
| Race/Ethnicity | -1.03 | 1.80 | -4.60 | 2.53 | -0.57 | 0.567 |
| Instagram daily time | -0.27 | 0.43 | -1.13 | 0.59 | -0.62 | 0.533 |
| Followers | -0.00 | 0.00 | -0.00 | 0.00 | -1.51 | 0.131 |
| Word count | 0.00 | 0.32 | -0.62 | 0.64 | 0.03 | 0.976 |
| Comments count | 0.00 | 0.00 | -0.00 | 0.00 | 1.48 | 0.140 |
| Social anxiety x ICSW | -0.03 | 0.01 | -0.06 | 0.00 | -1.98 | 0.049 |
| Expression suppression x ICSW | 0.09 | 0.05 | -0.02 | 0.21 | 1.63 | 0.104 |
| Cognitive reappraisal x ICSW | 0.00 | 0.04 | -0.08 | 0.09 | 0.15 | 0.876 |

**References**

Boyd, R. L., Ashokkumar, A., Seraj, S., & Pennebaker, J. W. (2022). The development and psychometric properties of LIWC-22. Austin, TX: University of Texas at Austin. https://www.liwc.app

Jordan, K. N., Pennebaker, J. W., & Ehrig, C. (2018). The 2016 US presidential candidates and how people tweeted about them. *Sage Open*, *8*(3), 2158244018791218.

Newman, M. L., Pennebaker, J. W., Berry, D. S., & Richards, J. M. (2003). Lying words: Predicting deception from linguistic styles. *Personality and Social Psychology Bulletin*, *29*(5), 665–675. https://doi.org/10.1177/0146167203029005010
